# Supplementary material for: Multiple drivers of the COVID-19 spread: The roles of climate, international mobility, and region-specific conditions
Source: PLoS One. 2020 Sep 23;15(9):e0239385. doi: 10.1371/journal.pone.0239385 (PMC7510993; doi:10.1371/journal.pone.0239385)
Supplement: S1 Appendix — (DOCX) [file pone.0239385.s004.docx]

**Appendix S1 List of data sources for the COVID-19 cases**

Belgium.be (2020) Coronavirus COVID-19. <https://www.info-coronavirus.be/nl/news/>

Bolivia Segura (2020) Coronavirus Bolivia. <https://www.boliviasegura.gob.bo/>

Bundesregierung (2020) Aktuelle Zahlen aus Österreich finden sie im amtlichen Dashboard zu COVID-19. <https://www.sozialministerium.at/Informationen-zum-Coronavirus/Neuartiges-Coronavirus-(2019-nCov).html>

Code for Romania (2020) COVID-19 Stiri oficiale. <https://stirioficiale.ro/informatii>

CSSE, J. (2020). Novel coronavirus(covid-19) cases. GitHub Repository, <https://github>. com/CSSEGISandData/COVID-19

Digital Government Development Agency (2020) Daily COVID-19 report. <https://data.go.th/en/dataset/covid-19-daily>

Dipartimento della Protezione Civile. (2020) COVID-19 Italia - Monitoraggio della situazione. [COVID-19 Italy – monitoring the situation]. Rome: Dipartimento della Protezione Civile. <https://github.com/pcm-dpc/COVID-19/tree/master/dati-province>

Direção-Geral da Saúde (2020) Ponto de Situação Atual em Portugal. <https://covid19.min-saude.pt/>

Federal Office of Public Health FOPH (2020) New coronavirus: Current situation – Switzerland and international. <https://www.bag.admin.ch/bag/en/home/krankheiten/ausbrueche-epidemien-pandemien/aktuelle-ausbrueche-epidemien/novel-cov/situation-schweiz-und-international.html>

Folkhälsomyndigheten. Antal fall av covid-19 i Sverige. <https://experience.arcgis.com/experience/09f821667ce64bf7be6f9f87457ed9aa>

Gobierno de México (2020) Secretaría de Salud, Archivo, Prensa. <https://www.gob.mx/salud/es/archivo/prensa?idiom=es&order=DESC&page=1>

Government of Pakistan (2020) Coronavirus in Pakistan. <http://covid.gov.pk/>

Gugus Tugas Percepatan Penanganan COVID-19 (2020) Dashboard Pemantauan Kasus COVID-19. <https://bnpb-inacovid19.hub.arcgis.com/>

Instituto de Salud Carlos III (ND). Situación de COVID-19 en España.　<https://covid19.isciii.es/>

Islamic Republic News Agency (2020) Iran’s coronavirus toll update. <https://en.irna.ir/>

J.A.G JAPAN Corp (2020) Coronavirus COVID-19 Japan Case by Each Prefecture (2019-nCoV). <https://gis.jag-japan.com/covid19jp/>

Korean Centers for Disease Control and Prevention. Coronavirus Disease-19, Republic of Korea. <http://ncov.mohw.go.kr/>

Maria Souquett Gil (2020) Venezuela sin reportes de nuevos casos de COVID-19. <https://efectococuyo.com/coronavirus/venezuela-sin-reportes-de-nuevos-casos-de-covid-19>

Ministério da Saúde (2020) Covid19: Painel Coronavirus. <https://covid.saude.gov.br/>

Ministerio de Salud (2020) Casos confirmados en Chile COVID-19. <https://www.minsal.cl/nuevo-coronavirus-2019-ncov/casos-confirmados-en-chile-covid-19/>

Ministerio de Salud (2020) Nuevo coronavirus COVID-19: Informe diario. <https://www.argentina.gob.ar/coronavirus/informe-diario>

Ministerio de Salud (2020) Situacion Nacional COVID-19. <https://www.ministeriodesalud.go.cr/index.php/centro-de-prensa/noticias/741-noticias-2020/1532-lineamientos-nacionales-para-la-vigilancia-de-la-infeccion-por-coronavirus-2019-ncov>

Ministerstwo Zdrowia (2020) Ministerstwo Zdrowia. <https://twitter.com/MZ_GOV_PL/status/1245431656380608518>

Ministry of Health – Manatū Hauora (2020) COVID-19 - current cases. <https://www.health.govt.nz/our-work/diseases-and-conditions/covid-19-novel-coronavirus/covid-19-current-situation/covid-19-current-cases>

Ministry of Health and Family Welfare. Government of India. (2020) COVID-19 in India. <https://www.mohfw.gov.in/>

Public Health England (2020) Coronavirus (COVID-19) cases in the UK. <https://coronavirus.data.gov.uk/>

publique France, S. (2020) Infection au nouveau coronavirus (SARS-CoV-2), COVID-19, France et Monde. <https://www.santepubliquefrance.fr/maladies-et-traumatismes/maladies-et-infections-respiratoires/infection-a-coronavirus/articles/infection-au-nouveau-coronavirus-sars-cov-2-covid-19-france-et-monde>

Robert Koch Institut (RKI) (2020) COVID-19: Fallzahlen in Deutschland und weltweit. https://www.rki.de/DE/Content/InfAZ/N/Neuartiges_Coronavirus/Fallzahlen.html

VG (2020) Live: Corona-viruset sprer seg i Norge og verden. <https://www.vg.no/spesial/2020/corona/>

Vlada Republike Hrvatske (2020) Koronavirus.hr. <https://www.koronavirus.hr/>

Министерстве здравоохранения Российской Федерации (2020)Коронавирус – симптомы, признаки, общая информация, ответы на вопросы — Минздрав России. <https://covid19.rosminzdrav.ru/>
