# Supplementary material for: Multiple drivers of the COVID-19 spread: The roles of climate, international mobility, and region-specific conditions
Source: PLoS One. 2020 Sep 23;15(9):e0239385. doi: 10.1371/journal.pone.0239385 (PMC7510993; doi:10.1371/journal.pone.0239385)
Supplement: Attachment — Submitted filename: Reviewer-commentsPONE-D-20-14796- Multiple Drivers.docx [file pone.0239385.s009.docx]

**Title:	Multiple drivers of the COVID-19 spread: role of climate, international mobility, and region-specific conditions**

**Authors;** Yasuhiro Kubota1*, Takayuki Shiono1, Buntarou Kusumoto2, Junichi Fujinuma1

**Manuscript Number:** PONE-D-20-14796

**Article Type: Research**

**Reviewer; Mohamed A Daw, *MD, PhD, MPS, FTCDI***

**Major Comments;**

**I read with great interest this paper and despite a lot of hypothetical assumption and ecological and bio epidemiological factors which is indeed difficult to calculate, I think the paper highlights and inspires a lot of new aspects that to be reflected on the global control of pandemic.**

**The paper need to be edited and formulated according the guide lines of the Journal-PLOS ONE**

**DECISION**

**ACCEPT,**
